# Exploring the Motivational Landscape: A Qualitative Analysis of Staff Empowerment in Hospital Settings

**DOI:** 10.7759/cureus.81754

**Published:** 2025-04-05

**Authors:** Abhijit Ravindra Chandankhede, Snehal D Thombre

**Affiliations:** 1 Neurosurgery, Shree Siddheshwar Multispeciality Hospital, Dhule, IND; 2 Anaesthesiology, Shree Siddheshwar Multispeciality Hospital, Dhule, IND

**Keywords:** ability, hospital staff, motivation, motivation factors, performance motivation, work efficiency

## Abstract

Background: Employee motivation is a critical determinant of hospital success, influencing productivity, job satisfaction, absenteeism, and turnover. In the healthcare sector, a highly motivated workforce enhances patient outcomes, fosters teamwork, and drives innovation. Understanding the factors that influence motivation is essential for developing an effective motivational program.

Aim: This study aims to evaluate the motivation levels of hospital employees and develop a comprehensive motivational program to enhance organizational performance.

Methodology: A cross-sectional study was conducted in selected hospitals, utilizing structured questionnaires and interviews to assess key motivational factors, including financial incentives, leadership styles, work environment, and career growth opportunities. Data were analyzed using statistical methods to identify trends and correlations.

Results: The findings reveal that financial incentives, professional development opportunities, and supportive leadership significantly enhance employee motivation. Additionally, a positive organizational culture and recognition programs play a vital role in improving job satisfaction and reducing turnover.

Conclusion: Hospitals must implement tailored motivational strategies to foster employee engagement and performance. Establishing a supportive work culture, providing career advancement opportunities, and recognizing employee contributions are crucial in enhancing motivation and ensuring high-quality healthcare delivery.

## Introduction

Background

Employee motivation plays a crucial role in organizational success, especially in healthcare, where staff efficiency directly impacts patient care quality. A well-designed motivational program enhances job satisfaction, productivity, and retention while reducing absenteeism and turnover [1]. In today's competitive environment, hospitals must foster motivated employees to maintain high-quality healthcare standards. According to Kinicki and Fugate [2], maintaining employee motivation is a challenging yet essential management task, particularly in a diverse and evolving workforce.

The Impact of Motivation on Hospital Success

Motivated employees are key to a hospital's success. Their drive influences productivity, attendance, and overall efficiency, directly affecting patient outcomes and satisfaction [3]. A strong motivational framework ensures that employees remain engaged, reducing errors and enhancing care delivery.

The Role of Motivated Employees in Hospital Performance

Employee motivation plays a crucial role in various hospital functions, including enhanced patient outcomes, increased productivity, and improved teamwork [4]. Motivated healthcare professionals provide better care, leading to faster patient recovery and higher satisfaction. Engaged employees perform tasks more efficiently, optimizing hospital operations, while a motivated workforce fosters collaboration and better communication, ultimately improving patient safety. Additionally, satisfied employees are less likely to leave or be absent, reducing hiring and training costs [5]. Motivated staff are also more likely to embrace innovation and adapt to new technologies and medical practices. Overall, enthusiastic employees contribute to a positive organizational culture, creating a supportive work environment and boosting overall morale.

Understanding Employee Motivation in Hospitals

Hospital managers must recognize that motivation varies among employees. Tailored strategies aligned with personal goals and workplace expectations foster a more engaged workforce. A structured approach to motivation enhances job satisfaction and performance.

The Complexity of Motivating Employees

Motivating employees is a complex task influenced by both intrinsic and extrinsic factors, including recognition, personal goals, and organizational culture. Developing an effective motivational program requires a deep understanding of these elements.

Knowledge gap

Despite extensive research on workplace motivation, limited studies focus specifically on hospital employees, who face unique challenges such as high stress, long hours, and emotional demands. Understanding how motivation strategies impact healthcare professionals is essential for improving hospital performance and patient care. This study aims to bridge this gap by identifying key motivational factors and proposing a structured framework for hospital management.

Objectives of the study

The objectives of this study are twofold: first, to evaluate the motivation levels of employees within the hospital and gain a comprehensive understanding of the factors that drive motivation in the healthcare context. This evaluation will involve assessing current motivational practices, identifying strengths and weaknesses, and exploring the individual needs, aspirations, and values that influence employee motivation. Second, to develop a tailored motivational program that addresses the identified needs and promotes high levels of employee engagement, job satisfaction, and performance. Drawing upon scientific theories of motivation and best practices from organizational psychology, this program aims to foster a motivating work environment that supports the growth and success of the hospital. This study provides valuable insights into the key factors influencing employee motivation in hospitals, with the ultimate goal of enhancing hospital performance and success. By fostering a supportive work environment, it encourages hospitals to nurture motivated employees, leading to improved patient care, organizational growth, and long-term sustainability.

## Materials and methods

This study used a qualitative method with the goal of identifying the factors that motivate workers in the hospital sector of Dhule, Maharashtra. Since this research aims to collect expressive information that cannot be conveyed through quantitative data, the qualitative approach was chosen as it is more appropriate. Purposive sampling was used to select the respondents for this study, who are workers in various positions at three hospitals in Dhule, Maharashtra, India, within a rural hospital setup.

By adopting a qualitative methodology, the study aimed to delve into the subjective experiences, perceptions, and motivations of hospital workers in Dhule, Maharashtra. It sought to understand the underlying factors that drive their motivation, such as job satisfaction, recognition, career advancement opportunities, work-life balance, and the impact of organizational culture. Through in-depth interviews, focus groups, or other qualitative data collection methods, the study aimed to gain a holistic understanding of the motivational factors specific to the hospitality sector in Dhule, Maharashtra.

The qualitative approach chosen for this study allows for a nuanced exploration of the workers' experiences, motivations, and perceptions. It provides an opportunity to capture the depth and richness of their perspectives, enabling a comprehensive understanding of the factors that contribute to their motivation. Ultimately, the findings of this qualitative research will contribute valuable insights to the field of hospitality management, informing strategies and practices aimed at enhancing employee motivation and job satisfaction in Dhule, Maharashtra.

A total of eight respondents participated in the research. A standardized structured interview method was used to collect data from the respondents, which took about two months to complete. The structured interview consists of several key questions that help define the areas to be explored, which are elements of motivation, and also allow the researcher to diverge to continue the idea or response for more details. The predominant interview format utilized by interviewers seeks to extract more comprehensive data from the respondents. Since the employees come from various work positions, this method is suitable to identify the elements of motivation among the employees. Data that have been collected in the research were analyzed using thematic analysis. Thematic analysis is appropriate for research that seeks to ascertain using interpretations and offered ordered elements to data analysis [6].

## Results

This section delves into the results obtained from the interview sessions conducted during the qualitative research phase, focusing on the various elements that contribute to employee motivation. The demographic profile of respondents is shown in Table 1.

**Table 1:** Age distribution of respondents

Respondent	Age
1	25
2	28
3	32
4	35
5	27
6	30
7	29
8	33

**Figure 1:**
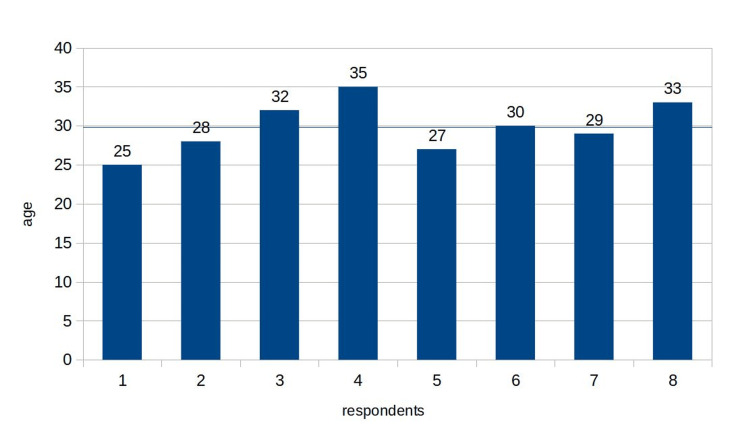
Age distribution of respondents

**Figure 2:**
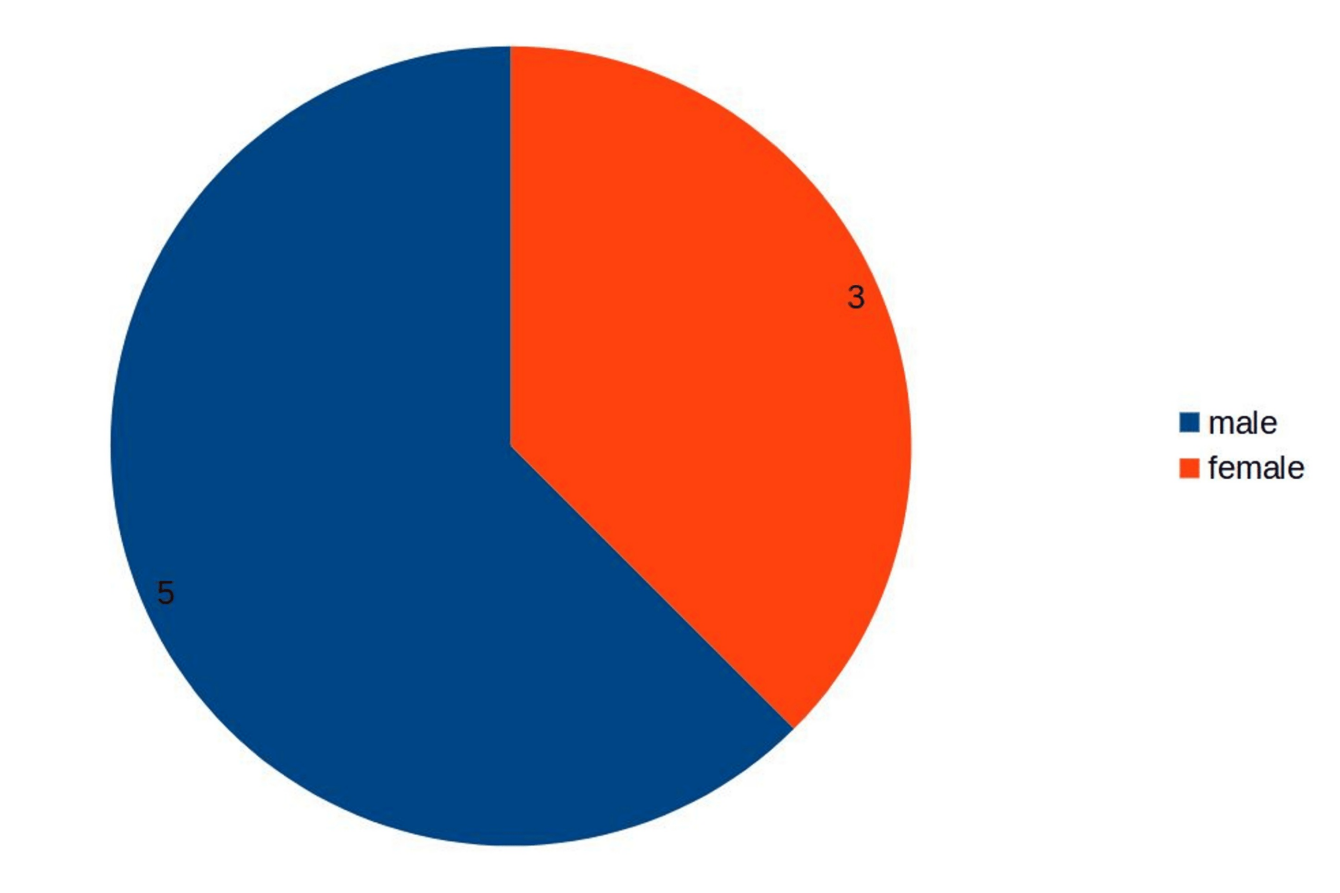
Gender distribution among the respondents

The mean age of the respondents was 29.875 with a standard deviation of 2.148 (Figure 1). Out of the eight respondents, five (62.5%) were male and three (37.5%) were female (Figure 2).

Through the investigation, the researcher identified nine distinct elements that significantly impact employee motivation. These elements include convenience, happiness, the working environment, personal will and desire, relationships with colleagues, work interest, salary, the relationship with the employer, and the opportunity to gain new knowledge. Each of these factors represents a unique aspect that influences the motivation levels of employees, as shown in Table 2.

**Table 2:** Summary of analysis of motivation elements among employees

Elements of motivation	S1	S2	S3	S4	S5	S6	S7	S8
Convenience	Stays in Dhule. Doesn’t feel like going anywhere	Stays in nearby village	Stays in the campus	Nearby village	Stays in Dhule, not far from hospital	Stays in the campus	Not stated	Stays in the campus
Happiness	Feels really happy	Feels happy to work with this hospital	Happy to work with this organization	Fells really happy	Feels happy to work with this hospital	Feels happy to work with this hospital	Feels happy to work with this hospital	Feels happy to work with this hospital
Working environment	Comfortable in the hospital	Feels safe and comfortable	Can work in the hospital without pressure	Feels motivated in the hospital environment	The workplace is very comfortable	Feels motivated to work here	Feels safe and comfortable	Feels safe and comfortable
Will and desire	Motivated to come to work because of the will and desire that comes from our self	Not stated	Not stated	Not stated	Own will to work in the field	Motivated by coworkers to work	Self-motivated due to desire to work in the field	Not stated
Relationship with colleagues	Healthy relationship that influences motivation	Feels happy to work with colleagues	Colleagues now became friends that motivate to work better	Friends that help each other and good understanding	Feel happy to work in the organization because of friends that give support	Friends that feel like siblings	Feels happy to work with colleagues	Feels happy to work with colleagues
Working interest	Not stated	Feels very interested in the job	Feels interested	Not stated	Not stated	Feels interested	Feels interested	Feels interested
Salary	Not stated	Good salary keeps me motivated	More salary would motivate more	Not stated	Not stated	Salary can affect the motivation to work	Good salary keeps me motivated	Good salary keeps me motivated
Relationship with the employer	Good and encouraging relations	Good relation	Motivating relations	Employer being very nice thus feels motivated	Employers always care about the workers	Employers always care about the workers	Good relation	Good and encouraging relation
Gaining knowledge	Get to learn new things	Knowledge keeps me motivated	Gaining more knowledge with each new patient	Learning new things everyday	Learning keeps me motivated	Not stated	Not stated	Learning new things keeps me motivated

Convenience refers to factors such as flexible working hours or convenient commuting options that contribute to an employee's overall motivation. Happiness encompasses a sense of satisfaction, joy, and contentment derived from one's work. The working environment, including factors like supportive culture and physical surroundings, has a direct impact on employee motivation.

Personal will and desire reflect the intrinsic motivation that arises from an individual's own aspirations and goals. Relationships with colleagues, characterized by teamwork, collaboration, and positive interactions, significantly influence employee motivation. Work interest refers to the level of engagement and enthusiasm an employee has toward their tasks and responsibilities.

The salary component is a crucial extrinsic motivator that influences employee motivation. The relationship with the employer, including factors like trust, respect, and effective communication, plays a vital role in shaping motivation levels.

Finally, the opportunity to gain new knowledge and skills through training and development initiatives is seen as a motivating factor for employees.

Understanding and addressing these nine elements of employee motivation are essential for organizations to create a conducive work environment that fosters high levels of motivation and engagement. The findings obtained from the qualitative research phase provide valuable insights into these elements and lay the foundation for further analysis and the development of strategies to enhance employee motivation within the context of the study.

Convenience

The ease and flexibility of work arrangements, including aspects such as flexible working hours, remote work choices, and easily accessible amenities, is referred to as convenience. Employees who have suitable work arrangements can attain a better work-life balance and enjoy lower stress levels, which improves their motivation.

Happiness

Happiness at work refers to employees' subjective experiences with positive feelings, job satisfaction, and fulfillment. Employees that are happy are more engaged, productive, and committed to their jobs. A positive work atmosphere, acknowledgment of accomplishments, and opportunities for personal growth and development all contribute to employee happiness.

Working environment

The physical, social, and cultural circumstances of the workplace are all part of the working environment. A comfortable and well-equipped office, supportive and collaborative relationships among coworkers, and a culture that values open communication, respect, and teamwork are all examples of pleasant working environments. A pleasant working environment promotes employee motivation by instilling a sense of belonging, trust, and psychological safety.

Personal will and desire

Will and desire refer to an individual's internal motivation, which is driven by personal objectives, goals, and passions. Employees are more likely to display high levels of commitment, initiative, and dedication to their work when they are motivated by their own inner drive and passion.

Relationship with coworkers

Positive relationships with coworkers are essential for employee motivation. When employees establish supportive, courteous, and collaborative relationships with their coworkers, it increases their sense of belonging and camaraderie develops teamwork and generates a pleasant social environment that adds to motivation and job satisfaction.

Work interest

Work interest refers to an employee's level of involvement, excitement, and satisfaction with their work and responsibilities. Employees are more likely to be motivated, perform at better levels, and feel more job satisfaction when their work is exciting, challenging, and matched with their talents and interests.

Salary

The extrinsic motivator of financial remuneration is salary. A fair and competitive wage is vital for motivating employees because it gives them a feeling of stability, rewards their efforts, and recognizes their importance to the organization.

Employee-employer connection

The relationship between employees and their employers includes elements such as trust, respect, effective communication, and management assistance. A great connection with the employer helps to motivate employees by instilling a sense of loyalty, dedication, and job satisfaction.

Gaining new knowledge

Employees are motivated when they have the opportunity to obtain new information, skills, and competencies through training and development activities. When workers have the opportunity to further their professional development and broaden their talents, it enhances their confidence, job satisfaction, and motivation, while also helping the organization by resulting in a more competent and capable workforce.

**Figure 3:**
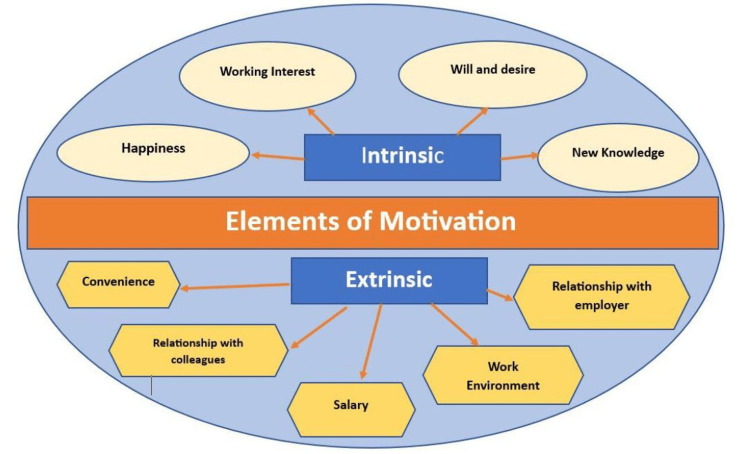
Elements of motivation among employees Image credits: Authors

In order to provide a more comprehensive understanding of the elements that drive motivation, these components are visually represented and depicted in Figure 3. The elements have been categorized into two main aspects: intrinsic motivation and extrinsic motivation. This division facilitates organizational comprehension of the motivational elements, enabling them to strategize their approaches and effectively maintain the motivation of their employees.

The depiction in Figure 3 serves to highlight the key elements that contribute to intrinsic motivation, which originates from internal factors such as personal fulfillment, passion, and the pursuit of individual goals. Additionally, it illustrates the elements associated with extrinsic motivation, which stem from external factors like rewards, recognition, and career advancement opportunities provided by the organization.

By visually breaking down the motivational elements into these distinct categories, organizations can gain a better understanding of the intricate dynamics of motivation. This understanding allows them to tailor their methods and implement targeted strategies to foster and sustain employee motivation. By comprehensively grasping various factors that influence motivation, organizations can create an environment that promotes employee engagement, productivity, and overall satisfaction.

## Discussion

The current research on employee motivation in the healthcare industry will be reviewed in the study's literature review. To comprehend the elements impacting motivation, the impact of motivation on hospital performance, and effective motivational practices applied in comparable contexts, it will investigate numerous theories, models, and empirical investigations.

The concept of employee motivation

According to Hodgetts and Hegar, motivation can be defined as a group of processes that provide support for individuals to engage in actions directed toward specific objectives [7]. Their perspective emphasizes the importance of exploring both the reasons behind and the mechanisms by which individuals' actions are influenced. In line with this view, Armstrong defines motivation as the factors that impact people's inclination to take specific actions. He further asserts that motivated individuals are more likely to move in a direction that leads to the attainment of predetermined goals. Moreover, Armstrong [8] presents a significant finding in the literature: individuals tend to repeat actions that have successfully fulfilled their needs. This emphasizes the crucial role of organizations in identifying the specific needs of employees to understand what motivates them to take the necessary steps. By comprehending these individual needs, organizations can tailor motivational strategies that align with employees' aspirations and foster a sense of fulfillment. This understanding allows for the creation of a work environment that not only addresses employees' needs but also encourages sustained motivation, engagement, and productivity. Effectively meeting these individual needs can improve job satisfaction, higher performance levels, and overall organizational success.

In essence, these perspectives highlight the significance of understanding motivation as a driving force for human behavior. By recognizing and addressing the underlying needs and factors that influence motivation, companies can foster a work environment that promotes employee engagement and achievement. This understanding underscores the importance of conducting research on motivation within organizations, such as hospitals, as it enables a deeper comprehension of the intricacies of employee motivation. By focusing on the "why" and "how" of what influences individuals' actions, organizations can develop effective strategies to motivate their employees and drive them toward accomplishing organizational objectives.

For hospitals, where the performance and well-being of employees have a direct impact on patient care and overall success, understanding employee motivation becomes even more crucial. By identifying and addressing the unique needs and aspirations of healthcare professionals, hospitals can create a motivating work environment that nurtures their dedication, enhances job satisfaction, and ultimately improves the quality of care provided.

Therefore, in the context of the proposed study titled "Motivation Practice for Hospital Employees," it is vital to delve into the complexities of employee motivation within the healthcare setting. By considering the perspectives of Hodgetts and Hegar [7] and Armstrong [8], the study can explore the underlying factors that drive motivation among hospital employees and develop targeted motivational programs to foster engagement, satisfaction, and optimal performance.

According to Bruce [9], there is a connection between employee motivation and performance, as the quality of the employees' work demonstrates their enthusiasm and passion for their tasks. According to Kroth [10], motivated employees are not always productive in line with their own internal motivation. This is due to the fact that motivation is influenced by a variety of different factors, including resources and a positive work environment. It goes without saying that managers must concentrate on both inner and extrinsic motivation because both have a significant impact on employees.

Theories of motivation

Theories of motivation include a variety of viewpoints that aim to explain why people act in particular ways. These theories include, among others, Herzberg's two-factor theory, Maslow's hierarchy of needs, and expectancy theory. They offer conceptual frameworks for comprehending the psychological, social, and cognitive aspects of motivation in humans.

Herzberg’s Two-Factor Theory

Herzberg's two-factor theory [11] proposes that there are two sets of factors influencing employee motivation and satisfaction. The "hygiene factors" are external to the job itself and include factors such as salary, job security, and working conditions. They are necessary to prevent dissatisfaction but do not directly contribute to motivation. The "motivational factors" are intrinsic to the job and include factors like recognition, growth opportunities, and challenging work. These factors directly influence motivation and job satisfaction, leading to increased productivity and engagement [12].

Herzberg's theory suggests that managers should focus on both hygiene factors and motivators in order to increase employee satisfaction and motivation. Hygiene factors should be kept at an adequate level in order to prevent dissatisfaction, but they will not lead to high levels of satisfaction on their own. Motivators, on the other hand, can lead to high levels of satisfaction if they are present [13].

McGregor Theory X and Theory Y

McGregor's theories X and Y offer opposing perspectives on worker motivation and management strategies. According to theory X, workers are innately sluggish, dislike their jobs, and are in need of severe oversight and management. According to this viewpoint, external rewards and penalties serve as the primary sources of motivation. Contrarily, theory Y contends that workers are self-driven, naturally motivated, and interested in prospects for professional advancement. Managers who adopt Theory Y support empowering staff members, fostering a positive work atmosphere, and promoting freedom and accountability. This approach supports participative decision-making and employee involvement and acknowledges that intrinsic motivation plays a critical role in fostering employee engagement, creativity, and productivity [14].

Maslow’s Hierarchy of Needs Theory

Maslow's hierarchy of needs is a psychological theory [15] proposed by Abraham Maslow, which suggests that individuals have a hierarchy of needs that drives their behavior and motivation. The theory consists of five levels of needs arranged in a pyramid, with each level building upon the previous one.

At the base of the pyramid are the physiological needs, such as food, water, shelter, and sleep. Once these basic needs are fulfilled, individuals strive for safety and security, including physical and emotional well-being. The next level is the need for love and belongingness, which involves forming meaningful relationships and being part of a social group.

Moving up the pyramid, the fourth level is the need for esteem, encompassing self-esteem and recognition from others. This includes the desire for achievement, respect, and status. Finally, at the top of the pyramid is self-actualization, representing the need for personal growth, self-fulfillment, and the realization of one's potential.

According to Maslow, individuals progress through these levels of needs, seeking to satisfy each level before moving on to the next. The theory suggests that once lower-level needs are met, higher-level needs become more salient and influential in driving behavior. However, it is important to note that not all individuals follow this exact hierarchical progression, as individual differences and cultural factors can influence the prioritization of needs.

Maslow's hierarchy of needs has been widely applied in various fields, including psychology, management, and education. It provides a framework for understanding human motivation and highlights the importance of fulfilling basic needs to foster well-being, engagement, and personal growth.

The Expectancy Theory of Motivation

The expectancy theory of motivation, proposed by Victor Vroom [16], suggests that individuals are motivated to act based on their expectations of the outcomes and their beliefs about the effort-performance-reward relationship. According to this theory, individuals are driven by three key factors: expectancy, instrumentality, and valence. Expectancy refers to the belief that effort will lead to performance, instrumentality relates to the belief that performance will result in desired outcomes or rewards, and valence pertains to the value or desirability of those outcomes. The theory posits that individuals are more motivated when they believe their efforts will lead to successful performance, that performance will be rewarded, and that the rewards are personally meaningful. Therefore, organizations should focus on strengthening these factors to enhance motivation, performance, and overall job satisfaction.

## Conclusions

Motivation is crucial for individual growth and organizational success. This study identifies five key motivational factors among hospitality employees: convenience, happiness, work environment, relationships, and interest. While motivation is influenced by internal drive, external factors like resources and workplace conditions also play a role.

To foster engagement and satisfaction, managers should address both intrinsic and extrinsic motivators. By applying these insights, organizations can create a supportive environment that enhances employee well-being and drives overall success.
